# Mapping condition-dependent regulation of metabolism in yeast through genome-scale modeling

**DOI:** 10.1186/1752-0509-7-36

**Published:** 2013-04-30

**Authors:** Tobias Österlund, Intawat Nookaew, Sergio Bordel, Jens Nielsen

**Affiliations:** 1 Novo Nordisk Foundation Center for Biosustainability, Chalmers University of Technology, Gothenburg, SE412 96, Sweden; 2 Novo Nordisk Foundation Center for Biosustainability, Technical University of Denmark, Hørsholm, DK2870, Denmark

**Keywords:** *Saccharomyces cerevisiae*, Genome-scale metabolic model, Integrated analysis, Transcriptionally controlled reactions

## Abstract

**Background:**

The genome-scale metabolic model of *Saccharomyces cerevisiae***,** first presented in 2003, was the first genome-scale network reconstruction for a eukaryotic organism. Since then continuous efforts have been made in order to improve and expand the yeast metabolic network.

**Results:**

Here we present iTO977, a comprehensive genome-scale metabolic model that contains more reactions, metabolites and genes than previous models. The model was constructed based on two earlier reconstructions, namely iIN800 and the consensus network, and then improved and expanded using gap-filling methods and by introducing new reactions and pathways based on studies of the literature and databases. The model was shown to perform well both for growth simulations in different media and gene essentiality analysis for single and double knock-outs. Further, the model was used as a scaffold for integrating transcriptomics, and flux data from four different conditions in order to identify transcriptionally controlled reactions, i.e. reactions that change both in flux and transcription between the compared conditions.

**Conclusion:**

We present a new yeast model that represents a comprehensive up-to-date collection of knowledge on yeast metabolism. The model was used for simulating the yeast metabolism under four different growth conditions and experimental data from these four conditions was integrated to the model. The model together with experimental data is a useful tool to identify condition-dependent changes of metabolism between different environmental conditions.

## Background

The number of metabolic network reconstructions for microorganisms has increased rapidly during the past decade following the genome revolution and the rapid increase of genome sequencing projects. The yeast *Saccharomyces cerevisiae* was the first eukaryote to have its metabolic network reconstructed and its metabolism is very well-studied. During the last 10 years the yeast metabolic network has been further updated and further reconstructed leading to several genome-scale models [1-9]. Yeast genome-scale modeling have many different applications, which have been reviewed lately [2]. Yeast has for example been used extensively in biotechnology as a workhorse for production of a wide range of chemicals, and metabolic modeling has helped in many cases to guide the strain construction by finding strategies for improved chemical production [10]. Recently the *S. cerevisiae* metabolic network has also been used as the basis for constructing metabolic models for other yeasts, e.g. *Schizosaccharomyces pombe*[11], *Yarrowia lipolytica*[12], *Pichia pastoris* and *Pichia stipitis*[13].

**Figure 1:**
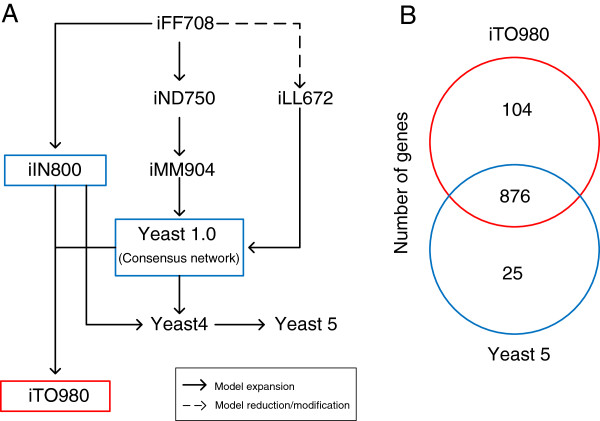
**The reconstruction process for iTO977 and its comparison with Yeast 5.** (**A**) Yeast genome-scale metabolic model pedigree. The iTO977 model (red) was reconstructed with the iIN800 model and the consensus network (blue) as starting points. The two starting point reconstructions were merged and further expanded by adding new pathways and reactions from databases and literature. (**B**) A comparison of the genes included in iTO977 and Yeast 5.

The first genome-scale metabolic model for *S. cerevisiae* was presented in 2003 [1] and since then no less than 11 genome-scale reconstructions have been released [2]. To overcome the problem with different models having different metabolite names, different scopes and representing parts of the metabolism in slightly different ways a consensus metabolic network was constructed as the result of a community jamboree effort [3]. The consensus network introduced a nomenclature standard for metabolites following MIRIAM, using ChEBI annotations and InChI codes. The consensus network can be viewed as a genome-scale reconstruction (GENRE) of the yeast metabolism, but not as a genome-scale model (GEM) ready for simulations using e.g. constraint-based methods such as flux balance analysis (FBA), due to missing information about reversibility of some reactions and missing biomass equations. The consensus network was further developed and improved and the updated version called Yeast 4 [4] is a GEM ready for simulations. It was further expanded and updated leading to another version called Yeast 5 [5]. The relationship between the yeast genome-scale models is shown in the pedigree in Figure 1A. The first model that included a detailed description of the lipid metabolism, namely iIN800 [8], was not included in the consensus reconstruction.

A genome-scale metabolic model can be thought of 1) being a biochemically and genetically structured database of the metabolism linking enzymes, metabolites and reactions together, and 2) a predictive model that can estimate the fluxes of intracellular reactions as well as predicting the growth of the cell when constraining the extracellular (measured) fluxes to represent the growth conditions. The first part corresponds to the GENRE and the second part corresponds to the GEM concept. The genome-scale metabolic model can be applied as an *in silico* hypothesis testing tool to gain insight into the operations of metabolism, leading to identification of targets for superior strain development.

Genome-scale metabolic model reconstructions can also be used as a scaffold for integrated data analysis, where the network structure of the model is the basis for the integration of omics data in order to gain more mechanistic knowledge of the cell’s behavior in different environments or under different conditions. The reporter metabolite algorithm [14] is a useful tool for integration of transcriptomics data into the metabolic network. The algorithm identifies key metabolites whose neighboring genes in the metabolic network are transcriptionally changed between two conditions. However, the regulation of the cellular response to different perturbations such as gene deletion or changes of environmental conditions may occur on many different levels in the cell. By using the genome-scale model to predict changes in metabolic flux between different conditions and correlate these changes with the change in expression of the genes involved, reactions that are directly controlled by transcription can be identified. The random sampling algorithm presented by Bordel et al. [15] is a framework for identification of this kind of trancriptionally controlled reactions using the genome-scale metabolic model.

Here we present an updated genome-scale model (GEM) that is based on the consensus network and the iIN800 model. The goal of the model is to provide an up-to-date and comprehensive description of the yeast metabolism, so the new model contains more genes, reactions and metabolites than previous models. We show that the model performs well in simulating the growth under different environmental conditions. The model was also used as a tool for integrating a large amount of omics data from different conditions. It was used together with the random sampling algorithm to correlate the changes in fluxes between aerobic and anaerobic conditions as well as carbon limited and nitrogen limited conditions with changes in gene expression in order to identify condition-dependent transcriptionally controlled reactions.

## Results and discussion

In order to construct a comprehensive genome-scale metabolic model, the consensus network was merged with the iIN800 model to construct a draft starting point reconstruction. The model was then improved by adding several new pathways, new reactions and new metabolites to the model based on database searches and literature evidence. The resulting model includes 977 metabolic genes and is called iTO977. Figure 1A shows how the iTO977 model was created. The model is available in SBML and Excel format (Additional files 1 and 2 and through the BioMet Toolbox [16], http://www.sysbio.se/biomet ) and a list of newly added reactions and pathways is available in Additional file 3.

### iTO977 model properties

We present an updated yeast model that is bigger in scope than earlier models; it contains more genes, metabolites and reactions than previous models. Table 1 shows a statistical summary of the current model and some previous models in terms of number of metabolites, genes and reactions. The iTO977 model has 4 compartments included in the model in contrast to the consensus network and Yeast 5 which both have 15 model compartments. The compartments in iTO977 are Cytoplasm, Mitochondria, Peroxisome and Extracellular. By keeping the number of compartments low the complexity of the model is reduced and its simulation capabilities are improved. Some of the reactions in the consensus network that takes place in other compartments, such as ER or nucleus, were included in the iTO977 model but localized to the cytoplasm, while some reactions in other compartments were out of scope of the model and discarded. The four compartments included in iTO977 are the compartments with the biggest confidence in terms of localization of the proteins in the Saccharomyces Genome Database (SGD, http://www.yeastgenome.org).

**Table 1:** Summary of the characteristics of different yeast genome-scale metabolic models

	**iFF708**	**iIN800**	**iMM904**	**Consensus**	**Yeast5**	**iTO977 (Current model)**
**Metabolites**	**825**	**985**	**1228**	**1168**	**1768**	**1353**
-Unique metabolites	595	683	713	664	779	815
-Cytosolic metabolites	518	645	634	590	579	738
-Mitochondrial mets	170	209	241	235	308	231
-Peroxisomal mets	0	0	80	80	75	87
-Extracellular metabolites	137	131	164	158	168	156
**Reactions**	**1145**	**1706**	**1577**	**1761**	**2034**	**1566**
-Unique reactions	780	1041	1053	804	1068	1207
-Cytosolic reactions	723	1301	710	835	1092	1083
-Mitochondrial reactions	104	242	306	330	334	256
-Peroxisomal reactions	0	0	109	121	111	80
**Genes**	**708**	**800**	**904**	**888**	**898**	**977**
-Percentage^1^	10.7%	12.1%	13.7%	13.4%	13.6%	14.8%
-Cytosolic genes	532	639	736	676	681	800
-Mitochondrial genes	104	124	199	190	204	212
-Peroxisomal genes	0	0	19	24	23	22

^1^ Part of the genome that is included in the model. Based on the updated statistics from Saccharomyces Genome Database that includes 6607 genes. (as of Aug 29, 2010).

When comparing the number of metabolites and reactions between different models it is important to keep in mind that the same metabolite can be present several times in the model, but in different compartments. In genome-scale metabolic models the same metabolite is considered as two different species if it is localized to two different compartments. When measuring the levels of intracellular metabolites by metabolomics it is impossible to distinguish metabolites in different compartments, e.g. pyruvate in the cytoplasm and pyruvate in mitochondria will be considered as the same species in a metabolome experiment. Similarly a reaction taking place in two different compartments will also be counted twice in the model. Since the yeast 5 model has many more compartments than iTO977 it also has a higher number of metabolites and reactions when taking compartmentalization into account. However, iTO977 has a higher number of unique metabolites and unique reactions than all previous models, i.e. without taking compartmentalization into account when counting the number of metabolites and reactions.

The number of genes included in the iTO977 model is larger than in previous models. Figure 1B shows the comparison of the iTO977 model and the Yeast 5 model in terms of number of open reading frames. A list of the ORFs included in the iTO977 model but not in Yeast 5 is presented in Additional file 4. The 25 ORFs that are in yeast 5 but not in iTO977 were all considered to be out of scope of the iTO977 model, since they are mainly transporters over e.g. the Golgi membrane, the vacuole membrane and other organelles that are not included in the iTO977 model. Among the 104 ORFs that are included in iTO977, but not in Yeast 5, many of them belong to two pathways that are newly introduced in the iTO977 model. These two pathways represent the biosynthesis of lipid-linked oligosaccharides [17] and Glycosylphosphaditylinositol [18], two molecules that are both used for protein modifications. These pathways provide a direct link between the iTO977 model and the recent model of the protein secretion machinery in yeast [19] and is a step towards connecting the metabolic model with a model for protein secretion.

### Model validation

The predictive power of the model was evaluated in two ways: 1) by comparing the simulated growth rate to experimental growth rate under different conditions, and 2) by comparing the predicted viability of *in silico* single and double gene knock-outs with the experimental observations of single and double mutant growth.

**Figure 2:**
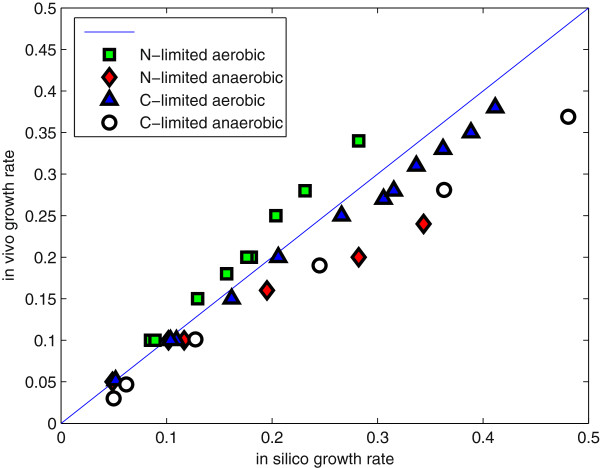
**Model validation by comparing *in silico *prediction of the specific growth rate with experimental data.** Growth phenotypes were collected from literature and compared to simulated values for chemostat cultivations at four different conditions, nitrogen limited aerobic (green) and anaerobic (red), carbon limited aerobic (blue) and anaerobic (white).

Figure 2 shows the results from the growth rate simulations. Experimental measurements for the specific growth rate, specific substrate consumption rate and specific product formation rate in chemostat cultures under four different conditions were taken from our previous study [20] and compared to simulated data using FBA (for more information, see materials and methods and Additional file 5). The four conditions that were considered were carbon limited aerobic growth, nitrogen limited aerobic growth, carbon limited anaerobic growth and nitrogen limited anaerobic growth. The model was shown to perform well in predicting the growth rate in all these four conditions.

**Figure 3:**
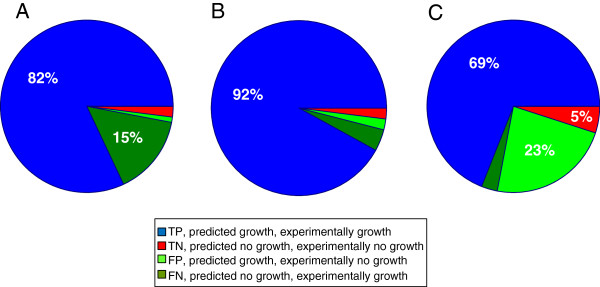
**Model validation by comparing simulation of single and double gene knock-out mutant growth to experimental observations.** (**A**) Single gene knock-out mutants cultivated in minimal media. (**B**) Single gene knock-out mutants cultivated in rich YPD media. (**C**) Double gene knock-out mutants cultivated in rich YPD media. TP = true positive, TN = true negative, FP = false positive and FN = false negative.

In order to evaluate the model’s capability of predicting gene essentiality we performed simulations of growth of single and double gene knock-outs. The viability of the mutants was compared to experimental observations from literature [6,21,22] and from SGD. Figure 3 shows the accuracy of the predictions for growth on glucose in minimal media (single gene knock-outs) and rich YPD media. A knock-out mutant was considered to be viable if the simulated growth rate of the mutant was within 10 percent of the wild type growth rate, otherwise the mutation was flagged as lethal. Simulations of growth phenotypes on other carbon sources such as ethanol and galactose and using different viability cut-offs are presented in Additional file 6.

### Integrated analysis

**Figure 4:**
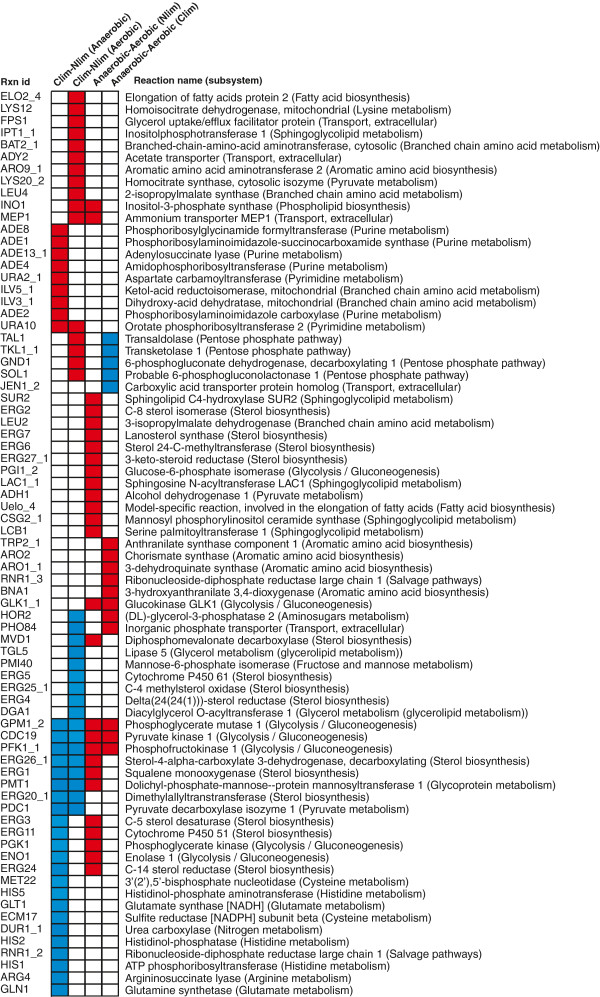
**Transcriptionally controlled reactions (reactions where the change in flux correlate with the change in expression of the involved genes) when comparing carbon limited with nitrogen limited growth and comparing aerobic and anaerobic growth.** Red color corresponds to an up-regulation both in flux and expression and blue color corresponds to down-regulation.

The iTO977 model was used as a scaffold for integrated data analysis. Chemostat cultivations were performed at a dilution rate of 0.05 h^-1^ under the same four different conditions as used for simulations above (carbon limited, nitrogen limited, aerobic and anaerobic) and microarray data as well as external and intracellular flux measurements was obtained for each condition [20]. We were particularly interested in changes that occur both on the transcript level and on the flux level, i.e. correlations between changes in transcriptome and fluxome under the different conditions. The enzymes that have a direct link between change in expression and change in flux might be good targets for over-expression in order to direct the flux toward i.e. a desired product, since there is a coupling between expression and flux. We therefore used the random sampling algorithm [15] to identify reactions that change both in flux and transcription. These reactions are referred to as transcriptionally controlled reactions and can be seen as a subset of the global set of reactions that change transcriptionally. The random sampling algorithm calculates a probability score for each reaction to change in flux between two conditions by using measured fluxes as additional constraints to the model to be able to simulate the two different conditions. The extracellular fluxes used to constrain the condition-specific models as well as the intracellular flux distributions predicted by each of the condition-specific models are presented in Additional file 7. The probability score of the reaction changing in flux is compared to the probability score of a transcriptional change of the involved genes, calculated from the microarray data. The transcriptionally controlled reactions when changing from carbon limited to nitrogen limited conditions or changing from aerobic to anaerobic conditions are shown in the heatmap in Figure 4. These reactions have a probability score calculated by the random sampling algorithm higher than 0.9, which indicates that the flux and transcript level for these reactions change in the same direction. Red color corresponds to up-regulation in both flux and in transcription, and blue corresponds to down-regulation both on the flux level and on the transcriptional level.

### Transcriptionally controlled reactions in glycolysis and ergosterol biosynthesis

**Figure 5:**
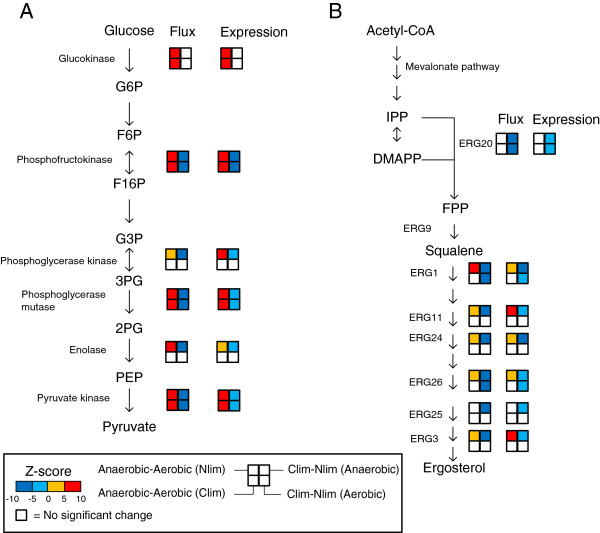
**Transcriptionally controlled reactions in glycolysis and around the FPP branch point.** (**A**) Three glycolytic reactions are considered to be transcriptionally controlled in all four conditions. The color indicates up- or down- regulation between two conditions, in flux and in expression. Only the reactions that are considered as transcriptionally controlled are shown. White color means that there was no significant change between the two conditions in that particular comparison. The four comparisons that were made was Anaerobic – Aerobic under nitrogen limitation (upper left corner), Anaerobic – Aerobic under carbon limitation (lower left corner), C-limited – N-limited under anaerobic conditions (upper right corner) and C-limited – N-limited under aerobic conditions (lower right corner). (**B**) Transcriptionally controlled reactions in the ergosterol biosynthesis pathway.

As expected we observe big differences in the glycolytic activity between anaerobic and aerobic chemostat growth and between carbon limited and nitrogen limited growth. There are three reactions that are considered to be transcriptionally controlled in all four comparisons (Figure 4), namely phosphofructokinase, phosphoglycerase mutase and pyruvate kinase. These reactions are all glycolytic reactions, and some of the other reactions in the glycolysis are also considered to be transcriptionally controlled, i.e. change in the same sense in flux and in expression (Figure 5A). The flux of these reactions and the expression of the involved enzymes are higher under anaerobic conditions than aerobic conditions. In the chemostat experiments the cells are controlled to grow with the same specific growth rate at all conditions. The cells have to consume more glucose under anaerobic conditions than under aerobic conditions in order to grow at the same rate, since anaerobic growth is less efficient energetically. Similarly, the glycolytic activtity is higher during nitrogen limited (glucose excess) conditions compared to carbon limited conditions, since there is more glucose available, and the cell uses more glucose to be able to produce both biomass and ethanol. The observation that the flux changes in the same direction as the expression of the enzymes catalyzing the reactions shown in Figure 5A suggests that the glycolytic flux is transcriptionally controlled through regulation of a few key enzymes when cell growth is constrained. This is in contrast to studies on engineering the glycolytic flux by over-expression glycolytic genes [23], which have shown that it is not possible to increase the glycolytic flux above the maximum observed at un-limited growth conditions.

Many of the steps in the ergosterol biosynthesis pathway were found to be down-regulated in both flux and expression in carbon limited conditions as compared with nitrogen limited conditions (Figure 5B). Many of the reactions were also up-regulated in both flux and expression in anaerobic conditions compared to aerobic under nitrogen limitation. Only the reactions considered to be transcriptionally controlled are presented in Figure 5.

### Identification of responsible transcription factors

In order to identify transcription factors responsible for the transcriptional control of the reactions shown in Figure 4 and Figure 5 we performed a hypergeometric enrichment test for each comparison and each transcription factor based on known transcription factor – gene interactions [24]. The transcription factors with a hypergeometric p-value less than 0.05 are shown in Table 2. The transcription factor Opi1p, a known negative regulator of phospholipid biosynthesis was over-represented in all four comparisons which suggest that the phospholipid metabolism is significantly changed in all four comparisons. Phospholipid synthesis is activated by the transcription factors Ino2p and Ino4p [25] and inactivated by Opi1p. Opi1p is in turn activated by protein kinase A (Tpk1p) that is activated at high glucose levels [26]. We found that most of the genes regulated by Opi1p are up-regulated in carbon limited conditions as compared to nitrogen limited conditions, which suggests that the flux through the phospholipid synthesis pathways is repressed by Opi1p at glucose excess conditions, but not at carbon limited conditions. We also found that most of the genes regulated by the Opi1p transcription factor are up-regulated at anaerobic conditions compared with aerobic conditions.

**Table 2:** Over-represented transcription factor interactions among significantly changed reactions

**Comparison**	**TF**	**Hypergeometric p-value**
Anaerobic - Aerobic (C-limited)	Opi1p	0.0009
	Pip2p	0.0168
	Gis2p	0.0278
Anaerobic - Aerobic (N-limited)	Opi1p	0.0215
C-limited - N-limited (Anaerobic)	Opi1p	0.0070
C-limited - N-limited (Aerobic)	Yap7p	0.0018
	Opi1p	0.0021
	Dal80p	0.0153
	Dig1p	0.0290

When comparing carbon limited growth with nitrogen limited growth during aerobic conditions we identify the transcription factors Opi1p, Yap7p, Dal80p and Dig1p as important regulators when considering both change in transcription and change in flux (Table 2). At glucose limited conditions yeast goes through full respiration and in glucose excess conditions the cell produces ethanol and the flux through the glycolysis is controlled by carbon catabolite repression. Similarly, at high nitrogen concentrations the nitrogen catabolite pathways are repressed [27]. The transcription factor Dal80p is known to be involved in nitrogen catabolite repression. In this analysis we find that the majority of the genes regulated by Dal80p have a higher expression and higher flux in carbon limited (excess of nitrogen) conditions than in nitrogen limited conditions under aerobic cultivation, which indicates that these pathways are transcriptionally regulated by Dal80p when changing from nitrogen limitation to carbon limitation.

## Conclusions

In this paper we present iTO977, a new updated genome-scale metabolic model for yeast. The model is bigger in scope than previous models and can be used both for simulations as well as a scaffold for integrated analysis. The model follows the standards that were introduced in the consensus reconstruction regarding annotation of metabolites and reactions and can therefore be seen as a well-annotated database representing the current knowledge of the yeast metabolism. By using the model to simulate different growth conditions and compare the simulated fluxes from different conditions using the random sampling algorithm we investigate the yeast metabolism in carbon limited, nitrogen limited, aerobic and anaerobic conditions. The change in predicted flux for each reaction in the model is compared with the changes in expression of the involved enzymes and reactions that are directly transcriptionally controlled are identified as well as transcription factors controlling these reactions. The genome-scale metabolic model together with experimental data is a valuable tool for data integration in order to find condition-dependent differences in yeast metabolism.

## Methods

### Model reconstruction

The first step in constructing the iTO977 model was to merge the iIN800 model [8] and the consensus network [3] to form a draft model. This was done by importing the SBML files for the two starting point reconstructions into Matlab format using the RAVEN toolbox [28]. Additional pathways and biochemical reactions were added from Kyoto Encyclopedia of Genes and Genomes [29], Saccharomyces Genome Database [30] and literature. A list of these reactions with references is available in Additional file 3. Reactions from the consensus network taking place in other compartments than extracellular, cytoplasm, mitochondria or peroxisome was moved to the cytoplasm before merging the two models. Since the iIN800 model doesn’t have peroxisome as a model compartment some of the reactions from iIN800 involved in fatty acid synthesis and degradation (beta-oxidation) was moved from cytoplasm to peroxisome before merging. Water and hydroxyl ions were not balanced in the model, assuming that there are other processes in the cell that uses these molecules as well. The model was corrected for inconsistencies and network gaps, using gap filling methods in RAVEN. The reactions identified as inconsistent by Zomorrodi et al. [31] were corrected to improve the ability to predict knock-out growth phenotypes. CHEBI identifiers and INCHI codes was added to all metabolites following the MIRIAM standard and the reactions in the model have links to EC-numbers or KEGG reactions. The biomass equation was taken from the iIN800 model. The final model was converted from Matlab format both to Excel format and SBML format.

### *In silico* growth simulations

Experimental data were collected from literature for chemostat cultivations performed at various dilution rates under each of the four different conditions: carbon limited aerobic [20,32-35], carbon limited anaerobic [36,37], nitrogen limited aerobic [35,36,38,39] and nitrogen limited anaerobic [40,41] (Additional file 5). For simulation purposes the uptake of glucose, NH_3_ and oxygen were constrained in the model according to the measured values for each experiment. FBA simulations were performed with maximization of biomass as the objective function and the deviation from the experimentally measured growth rate was identified.

Anaerobic conditions were simulated with the iTO977 model by constraining the oxygen uptake rate to zero, but also allowing for uptake of sterols to simulate that these compounds are supplied to the media for anaerobic growth.

### Gene essentiality predictions

*In silico* simulations of single gene knock-out growth were carried out for each of the genes in the model by constraining the flux of all corresponding reactions to zero and then estimating the maximal biomass flux using FBA. A relative fitness value was calculated for each mutant as f = mutant growth rate/wild type growth rate. A mutation was considered as lethal if the relative fitness was smaller than a cutoff value. Three different cutoffs were considered, 0.8, 0.9 and 0.95. The simulations were carried out for growth on glucose, both for a simulated minimal media, allowing only uptake of glucose, ammonium, oxygen, sulfate and phosphate, and for a simulated rich media (YPD media), where also uptake of amino acids and nucleotides was allowed. The viability of the mutants was compared to experimental observations of growth phenotypes for 524 single gene knockouts in minimal media and YPD media [6,30]. The model was also used to simulate the relative fitness for all pairs of double gene knock-outs, simulating growth on glucose in rich media (YPD) and compared to experimental observations for 234,719 double knock-outs [21].

### Random sampling

Experimental data from chemostat cultivations from four different conditions (carbon limited, nitrogen limited, aerobic and anaerobic) was taken from Jewett et al. [20]. The raw transcriptome data were normalized using the Probe Logarithmic Intensity Error (PLIER) method and the moderated t-statistic was used to identify significantly changed genes between two conditions. The upper and lower bounds of the exchange fluxes and lipid and biomass reactions in the iTO977 model were constrained according to experimentally measured fluxes (Additional file 7) for each condition. A set of flux distributions compatible with the measured fluxes was generated for each of the considered conditions as it has been previously described [15]. In order to avoid solutions involving loops we set the default bounds for the metabolic fluxes to Inf and –Inf instead of 1000 and −1000 as it is commonly done. For each pair of condition, we compared the first sample of the reference condition with all the samples of the second condition and computed how many times the reference had a higher (or lower) value for each reaction. The process was repeated for all the samples of the reference condition until all the possible combinations of samples at both conditions have been compared. The fraction of the comparisons in which the reference had a higher (or lower) value than the second condition was used as a probability score for the flux decreasing or increasing. The values of the moderated t-statistic from the transcriptome data were used to calculate a probability score P for each reaction representing the probability that the flux and the transcription is significantly changed in the same direction between two conditions. A hypergeometric enrichment test was performed to identify over-represented transcription factor interactions in the list of reactions from each comparison passing the cutoff P > 0.9 based on known transcription factor-gene interactions [24].

## Competing interests

The authors declare that they have no competing interests.

## Authors’ contributions

TÖ reconstructed the model, analyzed the data, performed simulations and performed the random sampling. SB was involved in manual curation of the model. IN, SB and JN designed the study and supervised the work. TÖ wrote the paper. All authors read and approved the final manuscript.

## Supplementary Material

Additional file 1 iTO977 model in Excel format including well-annotated spreadsheets for reactions, metabolites and genes. Click here for file

Additional file 2 **iTO977 model in SBML format.** The model is also available through the Biomet toolbox, http://www.sysbio.se/biomet. Click here for file

Additional file 3 Changes and additional reactions and pathways in iTO977. Click here for file

Additional file 4 ORFs not in iTO977 but in Yeast 5 and vice versa. Click here for file

Additional file 5 Growth simulation results. Click here for file

Additional file 6 Single and double growth knockout simulation results. Click here for file

Additional file 7 Additional constraints for condition-specific models used for random sampling. Click here for file
