# Supplementary material for: Mapping condition-dependent regulation of metabolism in yeast through genome-scale modeling
Source: BMC Syst Biol. 2013 Apr 30;7:36. doi: 10.1186/1752-0509-7-36 (PMC3648345; doi:10.1186/1752-0509-7-36)
Supplement: Additional file 3 — Changes and additional reactions and pathways in iTO977. [file 1752-0509-7-36-S3.pdf]

Additional file 3

Updates and new reactions and genes in iTO980 based on literature, KEGG and SGD

Lipid-linked oligosaccharide biosynthesis

New pathway in the iTO980 model, reference: Burda99a PMID: 9878760 [1]

ALG7, YBR243C ALG13, ALG14, ALG1, ALG2_1, ALG2_2, ALG11_1, ALG11_2, ALG3, ALG9_1, ALG12, ALG9_2, ALG6, ALG8, DIE2
Reference ALG13, ALG14 complex PMID: 16100110 [2]

Glycosylphosphatidylinositol (GPI) biosynthesis

New pathway in the iTO980 model, reference: PMID: 11356840 [3]

SPT14, GPI2, GPI3, GPI15 protein complex references PMID: 7768896 [4] and PMID: 11746600 [5],

Maranas' NGG inconsistencies

Updated reactions based on observations reported in Zomorrodi et al., PMID: 21190580 [6]

TPS1 (YBR126C) Change to reversible according to AZ. MALT is reversible based on ΔG. Hits found in yeast based on BLAST bi-directional test.

TPS2 (YDR074W) Change to reversible according to AZ. MALT is reversible based on ΔG. Hits found in yeast based on BLAST bi-directional test.

YJR073C Add new orf OPI3, as isoenzyme to the reaction YGR157W, CHO2, Reference PMID: 3066687 [7]

YPR058W, YBR104W (YMR1 and YMR2), isoenzymes to mitochondrial transporter proteins ODC1 and ODC2. Reference PMID:

KEGG

New genes and reactions added from KEGG pathways

CAB5, YDR196C Annotates reaction U96_ based on homology with KEGG.

SRY1, YKL218C, Serine racemase PMID: 12951240 [8]

Add transport reaction + exchange reaction of D-serine

OXP1, YKL215C PMID: 20402795 [9]

CAX4, YGR036C, PMID: 10024662 [10]

ALG5 YPL227C PMID: 8076653 [11] Irreversible reaction according to metacyc.

PGC1 YPL206C PMID: 18434318 [12] reaction mechanism in the paper

SGD

Added reactions and genes based on SGD pathways

GUD1 (YDL238C) Add according to SGD pathway, reversible, PMID: 15565584 [13]

EDH3 (YDR036C) Add according to SGD pathway, irreversible, PMID: 12697341 [14]

Coenzyme A biosynthesis

Based on paper about CoA biosynthesis in yeast, PMID: 19266201[15]

Annotate YDR531W as CAB1, add reaction CAB2 (YIL083C), annotate YKL088W (CAB3), YGR277C (CAB4) and YDR196C (CAB5). PMID: 19266201 [15]

gene CAB3 YKL088W works as a complex together with *SIS2(YKR072C) and VLH3 (*YOR054C) PMID: 19915539 [16]

Methionine salvage pathway,

New pathway based on evidence in literature. Reference PMID: 18625006 [17]

New reactions MRI1,MDE1,UTR4,ADI1,BAT2_4. UTR4 and ADI1 is lumped reaction in the consensus network. Split into two different reactions.. + include oxygen. PMID: 18625006 [17]

Quinon biosynthesis

CAT5 (YOR125C) New reaction from reference PMID: 8621692 [18]

Other changes

BUD16, YEL029C and BUD17 (YNR027W) Add reaction, isoenzymes. Pyridoxal phosphor transferase. Reference: PMID: 11452010 [19]

YEL042W extra orf, GDP phosphohydrolase, PMID: 7506254 [20]

NIT1, YIL164C Isoenzyme to NIT2. PMID: 11380987 [21]

PHO12, YHR215W isoenzyme to PHO11, PMID: 2646592 [22]

SOR2 isoenzyme to SOR1. PMID: 8125328 [23]

AAP1 (YHR047C) New reaction based on KEGG pathway. PMID: 8100228 [24]

APE2 (YKL158W) isoenzyme to AAP1. PMID: 6352682 [25]

YHR210C, isoenzyme to Gal10 PMID: 14764091 [26] sequence similarity confirmed by bbh blast

CTS1, CTS2 PMID: 1730413 [27]

GRE3 YHR104W, PMID: 11722921 [28]

LCS1 change to be complex with LCS1,LCS2 and YBR058C-A (TSC3) PMID:10713067 [29]

Csh1 added isoenzyme to SUR1 PMID: 12954640 [30]

Transporter YBR180W [31]

transporter mitochondria RIM2 YBR192W PMID: 7891656 [32]

YDL181W YDR322C-A added to the ATP synthase complex PMID: 16341776 [33]

YDL198C Mitochondrial ATP transport GCC1 PMID: 14998997 [34]

KCS1 added reaction according to iMM904

ATF2 isoenzyme to ATF1 PMID: 9836419 [35] (4 reactions)

References

1. Burda P, Aebi M: **The dolichol pathway of N-linked glycosylation**. *Biochimica et biophysica acta* 1999, **1426**(2):239-257.
2. Gao XD, Tachikawa H, Sato T, Jigami Y, Dean N: **Alg14 recruits Alg13 to the cytoplasmic face of the endoplasmic reticulum to form a novel bipartite UDP-N-acetylglucosamine transferase required for the second step of N-linked glycosylation**. *The Journal of biological chemistry* 2005, **280**(43):36254-36262.
3. Grimme SJ, Westfall BA, Wiedman JM, Taron CH, Orlean P: **The essential Smp3 protein is required for addition of the side-branching fourth mannose during assembly of yeast glycosylphosphatidylinositols**. *The Journal of biological chemistry* 2001, **276**(29):27731-27739.
4. Leidich SD, Kostova Z, Latek RR, Costello LC, Drapp DA, Gray W, Fassler JS, Orlean P: **Temperature-sensitive yeast GPI anchoring mutants gpi2 and gpi3 are defective in the synthesis of N-acetylglucosaminyl phosphatidylinositol. Cloning of the GPI2 gene**. *The Journal of biological chemistry* 1995, **270**(22):13029-13035.
5. Yan BC, Westfall BA, Orlean P: **Ynl038wp (Gpi15p) is the Saccharomyces cerevisiae homologue of human Pig-Hp and participates in the first step in glycosylphosphatidylinositol assembly**. *Yeast* 2001, **18**(15):1383-1389.
6. Zomorrodi A, Maranas C: **Improving the iMM904 S. cerevisiae metabolic model using essentiality and synthetic lethality data**. *BMC Systems Biology* 2010, **4**(1):178.
7. Summers EF, Letts VA, McGraw P, Henry SA: **Saccharomyces cerevisiae cho2 mutants are deficient in phospholipid methylation and cross-pathway regulation of inositol synthesis**. *Genetics* 1988, **120**(4):909-922.
8. Wada M, Nakamori S, Takagi H: **Serine racemase homologue of Saccharomyces cerevisiae has L-threo-3-hydroxyaspartate dehydratase activity**. *FEMS microbiology letters* 2003, **225**(2):189-193.
9. Kumar A, Bachhawat AK: **OXP1/YKL215c encodes an ATP-dependent 5-oxoprolinase in Saccharomyces cerevisiae: functional characterization, domain structure and identification of actin-like ATP-binding motifs in eukaryotic 5-oxoprolinases**. *FEMS yeast research* 2010, **10**(4):394-401.
10. van Berkel MA, Rieger M, te Heesen S, Ram AF, van den Ende H, Aebi M, Klis FM: **The Saccharomyces cerevisiae CWH8 gene is required for full levels of dolichol-linked oligosaccharides in the endoplasmic reticulum and for efficient N-glycosylation**. *Glycobiology* 1999, **9**(3):243-253.
11. Heesen S, Lehle L, Weissmann A, Aebi M: **Isolation of the ALG5 locus encoding the UDP-glucose:dolichyl-phosphate glucosyltransferase from Saccharomyces cerevisiae**. *European journal of biochemistry / FEBS* 1994, **224**(1):71-79.
12. Simockova M, Holic R, Tahotna D, Patton-Vogt J, Griac P: **Yeast Pgc1p (YPL206c) controls the amount of phosphatidylglycerol via a phospholipase C-type degradation mechanism**. *The Journal of biological chemistry* 2008, **283**(25):17107-17115.
13. Saint-Marc C, Daignan-Fornier B: **GUD1 (YDL238c) encodes Saccharomyces cerevisiae guanine deaminase, an enzyme expressed during post-diauxic growth**. *Yeast* 2004, **21**(16):1359-1363.
14. Hiltunen JK, Mursula AM, Rottensteiner H, Wierenga RK, Kastaniotis AJ, Gurvitz A: **The biochemistry of peroxisomal beta-oxidation in the yeast Saccharomyces cerevisiae**. *FEMS microbiology reviews* 2003, **27**(1):35-64.
15. Olzhausen J, Schubbe S, Schuller HJ: **Genetic analysis of coenzyme A biosynthesis in the yeast Saccharomyces cerevisiae: identification of a conditional mutation in the pantothenate kinase gene CAB1**. *Current genetics* 2009, **55**(2):163-173.

16. Ruiz A, Gonzalez A, Munoz I, Serrano R, Abrie JA, Strauss E, Arino J: **Moonlighting proteins Hal3 and Vhs3 form a heteromeric PPCDC with Ykl088w in yeast CoA biosynthesis**. *Nature chemical biology* 2009, **5**(12):920-928.
17. Pirkov I, Norbeck J, Gustafsson L, Albers E: **A complete inventory of all enzymes in the eukaryotic methionine salvage pathway**. *The FEBS journal* 2008, **275**(16):4111-4120.
18. Marbois BN, Clarke CF: **The COQ7 gene encodes a protein in saccharomyces cerevisiae necessary for ubiquinone biosynthesis**. *The Journal of biological chemistry* 1996, **271**(6):2995-3004.
19. Ni L, Snyder M: **A genomic study of the bipolar bud site selection pattern in Saccharomyces cerevisiae**. *Molecular biology of the cell* 2001, **12**(7):2147-2170.
20. Berninsone P, Miret JJ, Hirschberg CB: **The Golgi guanosine diphosphatase is required for transport of GDP-mannose into the lumen of Saccharomyces cerevisiae Golgi vesicles**. *The Journal of biological chemistry* 1994, **269**(1):207-211.
21. Pace HC, Brenner C: **The nitrilase superfamily: classification, structure and function**. *Genome biology* 2001, **2**(1):REVIEWS0001.
22. Venter U, Horz W: **The acid phosphatase genes PHO10 and PHO11 in S. cerevisiae are located at the telomeres of chromosomes VIII and I**. *Nucleic Acids Res* 1989, **17**(4):1353-1369.
23. Sarthy AV, Schopp C, Idler KB: **Cloning and sequence determination of the gene encoding sorbitol dehydrogenase from Saccharomyces cerevisiae**. *Gene* 1994, **140**(1):121-126.
24. Caprioglio DR, Padilla C, Werner-Washburne M: **Isolation and characterization of AAP1. A gene encoding an alanine/arginine aminopeptidase in yeast**. *The Journal of biological chemistry* 1993, **268**(19):14310-14315.
25. Trumbly RJ, Bradley G: **Isolation and characterization of aminopeptidase mutants of Saccharomyces cerevisiae**. *J Bacteriol* 1983, **156**(1):36-48.
26. Majumdar S, Ghatak J, Mukherji S, Bhattacharjee H, Bhaduri A: **UDPgalactose 4-epimerase from Saccharomyces cerevisiae. A bifunctional enzyme with aldose 1-epimerase activity**. *European journal of biochemistry / FEBS* 2004, **271**(4):753-759.
27. Dohrmann PR, Butler G, Tamai K, Dorland S, Greene JR, Thiele DJ, Stillman DJ: **Parallel pathways of gene regulation: homologous regulators SWI5 and ACE2 differentially control transcription of HO and chitinase**. *Genes & development* 1992, **6**(1):93-104.
28. Traff KL, Otero Cordero RR, van Zyl WH, Hahn-Hagerdal B: **Deletion of the GRE3 aldose reductase gene and its influence on xylose metabolism in recombinant strains of Saccharomyces cerevisiae expressing the xylA and XKS1 genes**. *Appl Environ Microbiol* 2001, **67**(12):5668-5674.
29. Gable K, Slife H, Bacikova D, Monaghan E, Dunn TM: **Tsc3p is an 80-amino acid protein associated with serine palmitoyltransferase and required for optimal enzyme activity**. *The Journal of biological chemistry* 2000, **275**(11):7597-7603.
30. Uemura S, Kihara A, Inokuchi J, Igarashi Y: **Csg1p and newly identified Csh1p function in mannosylinositol phosphorylceramide synthesis by interacting with Csg2p**. *The Journal of biological chemistry* 2003, **278**(46):45049-45055.
31. Felder T, Bogengruber E, Tenreiro S, Ellinger A, Sá-Correia I, Briza P: **Dtr1p, a multidrug resistance transporter of the major facilitator superfamily, plays an essential role in spore wall maturation in Saccharomyces cerevisiae**. *Eukaryotic cell* 2002, **1**(5):799-810.
32. Dyck E, Jank B, Ragnini A, Schweyen RJ, Duyckaerts C, Sluse F, Foury F: **Overexpression of a novel member of the mitochondrial carrier family rescues defects in both DNA and RNA metabolism in yeast mitochondria**. *Molecular and General Genetics MGG* 1995, **246**(4):426-436.
33. Contessi S, Haraux F, Mavelli I, Lippe G: **Identification of a conserved calmodulin-binding motif in the sequence of F0F1 ATPsynthase inhibitor protein**. *Journal of bioenergetics and biomembranes* 2005, **37**(5):317-326.

34. Vozza A, Blanco E, Palmieri L, Palmieri F: **Identification of the mitochondrial GTP/GDP transporter in Saccharomyces cerevisiae**. *The Journal of biological chemistry* 2004, **279**(20):20850-20857.
35. Nagasawa N, Bogaki T, Iwamatsu A, Hamachi M, Kumagai C: **Cloning and nucleotide sequence of the alcohol acetyltransferase II gene (ATF2) from Saccharomyces cerevisiae Kyokai No. 7**. *Bioscience, biotechnology, and biochemistry* 1998, **62**(10):1852-1857.
